# Diabetes is an independent predictor of right ventricular dysfunction post ST-elevation myocardial infarction

**DOI:** 10.1186/s12933-016-0352-2

**Published:** 2016-02-18

**Authors:** Idan Roifman, Nilesh Ghugre, Mohammad I. Zia, Michael E. Farkouh, Anna Zavodni, Graham A. Wright, Kim A. Connelly

**Affiliations:** Schulich Heart Program, Sunnybrook Health Sciences Centre, University of Toronto, Toronto, ON Canada; Physical Sciences Platform, Sunnybrook Health Sciences Centre, University of Toronto, Toronto, ON Canada; Department of Medical Biophysics, Sunnybrook Health Sciences Centre, University of Toronto, Toronto, ON Canada; Division of Cardiology, University Health Network, University of Toronto, Toronto, ON Canada; Keenan Biomedical Research Centre, Li Ka Shing Knowledge Institute, St. Michael’s Hospital, University of Toronto, Toronto, ON Canada

**Keywords:** Diabetes mellitus, Right ventricular dysfunction, ST elevation myocardial infarction, Cardiovascular magnetic imaging resonance

## Abstract

**Background:**

Diabetes mellitus (DM) is estimated to become the 7th leading cause of death by 2030. Right ventricular dysfunction (RVD) complicating ST elevation myocardial infarction (STEMI) is independently associated with a higher mortality; however the relationship between DM and RVD is currently unknown. The purpose of this study was to determine whether DM is an independent predictor for the presence of right ventricular dysfunction (RVD) post STEMI.

**Methods:**

106 patients post primary PCI for STEMI were enrolled in the study. Cardiac MRI was performed within 48–72 h after admission in order to assess ventricular function. Statistical analysis consisted initially of descriptive statistics including Chi square, Fisher’s exact, or the Wilcoxon rank sum as appropriate. Subsequently, logistic regression analysis was performed to determine independent predictors of RVD.

**Results:**

The median age in the study was 58 years (IQR 53, 67). 30 % of the patients had diabetes. Of 99 patients for which RV data was available, 40 had RVD and 59 did not. Patients with DM were significantly more likely to have RVD when compared to those without diabetes (45 vs 22 %, p = 0.03). There was no significant difference in age, hypertension, smoking status, dyslipidemia, serum creatinine or peak CK levels between the two groups. After adjusting for other factors, presence of DM remained an independent predictor for the presence of RV dysfunction (OR 2.78, 95 % CI 1.12, 6.87, p = 0.03). Amongst diabetic patients, those with HbA1C ≥ 7 % had greater odds of having RVD vs those with HbA1C < 7 % (OR 5.58 (1.20, 25.78), p = 0.02).

**Conclusions:**

The presence of DM conferred an approximately threefold greater odds of being associated with RVD post STEMI. No other major cardiovascular risk factors were independently associated with the presence of RVD.

## Background

Diabetes mellitus (DM) is major public health problem. The world health organization estimates that by 2030, 347 million people will be affected worldwide and it will be the seventh leading cause of death [1]. A major reason for the increased mortality is via the presence of coronary artery disease (CAD) [2–5]. Right ventricular myocardial infarction (RVMI) and subsequent dysfunction (RVD) are associated with a poor prognosis that is independent of left ventricular (LV) dysfunction [6]. Despite its clinical importance, little is currently known regarding the clinical predictors of right ventricular dysfunction post myocardial infarction (MI). Further, whether or not DM is a predictor for the presence of RVD is currently unknown. On one hand DM is a major cardiovascular risk factor and may predispose to more post-MI complications such as RVD. Further, evidence from studies examining diabetic cardiomyopathy suggest an extensive involvement of the microvasculature in the presence of this entity [7]. The right ventricle, being extensively perfused by the microvasculature [8, 9], may be more prone to microvascular injury. On the other hand, more extensive collateralization [10–13] in patients with DM may confer a relative protective effect. Thus, the purpose of this study is both to explore clinical predictors of RVD post MI and to determine if DM is an independent predictor for its presence. We hypothesized that the presence of diabetes would be an independent predictor for the presence of RVD post STEMI.

## Methods

### Patient population and data sources

Data was collected from 106 consecutive patients post STEMI who were prospectively enrolled in this study at Sunnybrook Health Sciences Centre between the years 2009–2013. Each patient had a research cardiac magnetic resonance (CMR) scan within 48–72 h of admission. The only inclusion criterion for this study was presentation with a STEMI. Exclusion criteria included presence of hemodynamic instability, requirement for urgent coronary artery bypass grafting, severe kidney disease as defined by an estimated glomerular filtration rate (eGFR) of <30 mL/min and failure to provide informed consent to participate in the study. All patients were successfully revascularized via primary percutaneous coronary intervention (including the deployment of coronary stents) and received current standard of care medications [14, 15]. This study was approved by the research ethics board of Sunnybrook Health Sciences Centre.

### CMR protocol

#### Cardiac MR acquisition protocol

Each patient was scanned at 48–72 h post primary PCI. All studies were performed on a GE 1.5 Tesla MR scanner (Signa Twinspeed HDx; GE Healthcare, Waukesha, WI) using an 8-channel receiver coil. RV function was determined using contiguous short axis slices covering the left and right ventricle acquired with a standard SSFP (FIESTA) sequence with the following parameters: echo time 1.6 ms, flip angle 45°, acquisition matrix 256 × 192, bandwidth 125 kHz, and 20 cardiac phases per slice.

### Cardiac MR image analysis

**Fig. 1:**
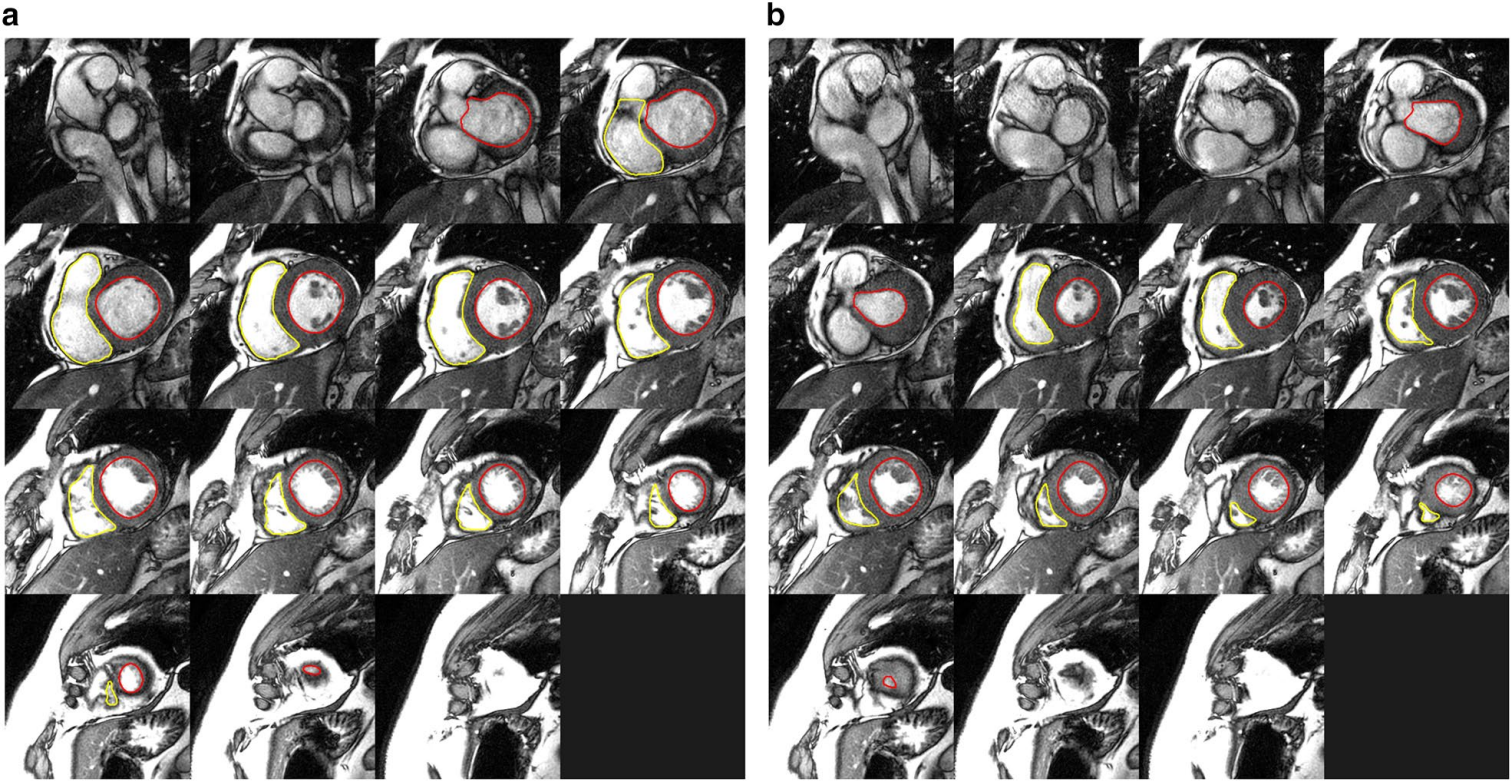
Example of the contouring of the left (*red*) and right (*yellow*) ventricular endocardium in one of the patients in our study during end-diastole (**a**) and end systole (**b**)

CVI-42 version 4.1.8 software (Circle Cardiovascular Imaging, Calgary, Alberta, Canada) was used to contour the left and right ventricles and derive volumes and ejection fractions. Studies were contoured by a level 3 Society for Cardiovascular Magnetic Resonance (SCMR) trained physician according to the most recent guideline recommendations [16]. Specifically, the endocardial contours of the left and right ventricle were manually traced at end-diastole and end-systole in all cine studies. For the right ventricle, the pulmonary valve was visualized and contours were included up to but not above this level. Trabeculations of the right ventricle were ignored and a smooth endocardial border was drawn for each slice (see Fig. 1). LV and RV stroke volumes were within 10 % of each other in the studies thus providing confirmation of our results (given that none of the patients had significant intra or extra-cardiac shunts).

### Predictors and outcome

The outcome of interest was presence or absence of RVD post STEMI. RVD was defined as RVEF <50 % [17–20]. The key predictor of interest was presence or absence of DM. DM status was determined via a combination of patient interview, chart review and assessment of hemoglobin A1c at the time of presentation with STEMI. The following co-variates were included in the study based on the fact that they have been shown to be predictors for the presence of CAD and MI: age, hypertension, prior MI, smoking status, hyperlipidemia status, peak creatine kinase (CK) levels and serum creatinine [21–23].

We first performed statistical analyses assessing the relationship between the predictors and the outcome. For categorical variables, 2 × 2 contingency tables were constructed. Subsequently, the Chi square test of association was used to compare predictors across the outcomes of interest. Where small numbers of sub-groups existed, Fisher’s exact test was utilized. Normally distributed continuous variables were compared using t-tests. Non-normally distributed continuous variables were compared with the Wilcoxon rank sum test. Normality of distribution was assessed using the Shapiro–Wilk test. Additionally, similar statistical analyses were used to compare characteristics of patients with and without diabetes. A logistic regression analysis was performed in order to examine if DM is an independent predictor for the presence of an RVMI after adjusting for co-variates. Prior to performing the logistic regression analyses, all predictor variables were assessed for the presence of multicollinearity using variance inflation factor and tolerance. LVEF was not included in the logistic regression models because it was found to be significantly co-linear with peak CK. A screening strategy for variable selection was selected utilizing a conservative *p* value of ≤0.25 to identify covariates to be used in the logistic regression model. Furthermore, we performed a subgroup analysis for those with HbA1C < 7 vs ≥7. HbA1C was determined by venous sampling and was performed based on clinical suspicion for the presence of diabetes. We also performed a sensitivity analysis using an RVEF cut-off value of 47 % for normality (as advocated by some investigators) [9]. All statistical analyses were done with SAS version 9.3 (Cary, North Carolina). Statistical significance was defined as a two-tailed p value of <0.05.

## Results

### Descriptive statistics

Table 1 displays the characteristics of the entire patient population consisting of 106 patients. Median age in the sample was 58 years (IQR 53, 67). Most patients were male (88.7 %). 51.8 % on the infarcts involved the left anterior descending (LAD) as the culprit vessel, 27.3 % the right coronary artery (RCA) and 17.9 % the left circumflex (LCx). 30.2 % of the patients had DM. In terms of other major cardiovascular risk factors, 44.3 % had hypertension, 41.9 % were active smokers and 32.1 % had dyslipidemia. The mean LVEF was 43.5 % and mean RVEF was 51.8 %. Seven patients were excluded due to poor visualization of the right ventricular endocardial contours on SSFP imaging. Table 2 displays the characteristics of the 99 patients who had diagnostic RV images for the determination of RVEF. These patient characteristics are similar to those of the original population of 106 patients. Table 3 displays characteristics of patients with and without diabetes mellitus. Of note, patients with diabetes were significantly more likely to have previous PCI (p = 0.03) and dyslipidemia (p = 0.001). There was a trend towards lower LVEF in diabetic patients (41.4 ± 7.8 % vs 44.3 ± 7.9 % in patients without diabetes, p = 0.09). There were no significant differences between the two groups in terms of other major cardiovascular risk factors, location of culprit vessel, presence of multi-vessel CAD or post PCI TIMI flow.

**Table 1:** Characteristics of the patient population

Total n = 106	
Age in years (median, IQR)	58 (53, 67)
Female, n (%)	12 (11.3)
Culprit vessel (%)	
LAD	58 (51.8)
LCx	19 (17.9)
RCA	29 (27.3)
Previous MI, n (%)	5 (4.7)
Diabetes, n (%)	32 (30.2)
Hypertension, n (%)	47 (44.3)
Smoking, n (%)	44 (41.9)
Dyslipidemia, n (%)	34 (32.1)
Previous PCI, n (%)	3 (2.8)
Previous CABG, n (%)	1 (0.9)
Serum creatinine in umol/l (median, IQR)	78 (67, 93)
Peak CK in standard units/l (median, IQR)	1788 (986, 2767)
LVEF, mean (SD)	43.45 (8.0) %
RVEF, mean (SD)	51.77 (9.1) %
RVD	40 (40.4)
Multi-vessel CAD	61 (57.6)
TIMI ≥2	105 (99.1)

Continuous variables that are not normally distributed are reported as median (IQR)

Continuous variables that are normally distributes are reported as mean (standard deviation)

Categorical variables are reported as frequencies and percentages

**Table 2:** Characteristics of the patient population who had diagnostic RV images for determination of RVEF

Total n = 99	
Age in years (median, IQR)	58 (52, 67)
Female, n (%)	10 (10)
Culprit vessel (%)	
LAD	52
LCx	19
RCA	28
Previous MI, n (%)	5 (5)
Diabetes, n (%)	31 (31)
Hypertension, n (%)	44 (44)
Smoking, n (%)	42 (42)
Dyslipidemia, n (%)	32 (32)
Previous PCI, n (%)	3 (3)
Previous CABG, n (%)	1 (1)
Serum creatinine in umol/l (median, IQR)	
Peak CK in standard units/l (median, IQR)	1815 (905, 2774)
LVEF, mean (SD)	43.26 (8.2)
RVEF, mean (SD)	51.77 (9.1)
RVD	40 (40)
Multi-vessel CAD (%)	57 (57)
TIMI ≥ 2	98 (98)

Continuous variables that are not normally distributed are reported as median (IQR)

Continuous variables that are normally distributes are reported as mean (standard deviation)

Categorical variables are reported as frequencies and percentages

**Table 3:** Characteristics of patients with and without diabetes mellitus

Total n = 106			
	DM (n = 32)	No DM (n = 74)	p value
Age in years (median, IQR)	59 (54, 67)	58 (53, 66)	0.84
Female sex (%)	2 (6.3)	10 (13.5)	0.34
Previous MI (%)	3 (9.4)	2 (2.7)	0.14
Previous PCI (%)	3 (9.4)	0 (0)	0.03
Previous CABG (%)	0 (0)	1(1.4)	0.99
Hypertension (%)	14 (43.8)	33 (44.6)	0.94
Smoking (%)	16 (50)	28 (38.4)	0.27
Hyperlipidemia (%)	16 (50)	18 (24)	0.001
Serum creatinine in umol/l (median, IQR)	75 (64, 94)	78 (61, 78)	0.64
Peak CK in standard units/l (median, IQR)	1838 (945, 2731)	1784 (997, 3276)	0.44
LVEF, mean (SD)	41.4 % (7.8)	44.3 % (7.9)	0.08
Culprit vessel (%)			0.19
LAD	16 (50)	42 (56.7)	
LCX	9 (28.3)	10 (13.5)	
RCA	7 (21.9)	22 (29.7)	
Multi-vessel CAD (%)	14 (43.8)	47 (63.5)	0.09
TIMI ≥2	31 (96.9)	74 (100)	0.21
HbA1C (SD)	7.5 (0.8)	5.4 (0.4)	<0.001

Table 4 compares patient characteristics between the RVD and the non RVD group. Out of the 99 patients, 40 had RVD and 59 did not. The mean RVEF for patients with RVD was 42.8 ± 5.1 % vs 57.9 ± 5.2 % for those without RVD (p < 0.001).

**Table 4:** Characteristics of patients with and without RVD

Total n = 99	RVD (n = 40)	No RVD (n = 59)	p value
Age in years (median, IQR)	58 (52, 68)	58 (53, 66)	0.71
Female sex (%)	5 (12.5)	5 (8.5)	0.52
Previous MI (%)	4 (10.0)	1 (1.7)	0.15
Previous PCI (%)	2 (5.0)	1 (1.7)	0.56
Previous CABG (%)	1 (2.5)	0 (0)	0.40
Hypertension (%)	16 (40.0)	28 (47.5)	0.46
Smoking (%)	17 (42.5)	25 (42.3)	0.91
Dyslipidemia (%)	14 (35.0)	18 (30.5)	0.64
Serum creatinine in umol/l (median, IQR)	78 (66.50, 88.50)	78 (67, 95)	0.75
Peak CK in standard units/l (median, IQR)	1666.5 (815, 2395)	2096 (1103, 2964)	0.25
LVEF, mean (SD)	43.5 (9.1) %	41.5(7.2) %	0.16
Culprit vessel (%)			0.41
LAD	20 (50)	32 (54)	
LCX	6 (15)	13 (22)	
RCA	14 (35)	14 (24)	
Multi-vessel CAD (%)	25 (62.5)	32 (54.2)	0.42
TIMI ≥2	40 (100)	58 (98.3)	0.79
Diabetes (%)	18 (45.0)	13 (22.0)	0.03

There was no significant difference between the RVD and the no RVD groups in terms of median age (58 vs 58 years, p = 0.71), female sex (8.5 vs 12.5 %, p = 0.52), hypertension (40 vs 47.5 %, p = 0.46), current smoking status (43.6 vs 42.4 %, p = 0.64) and serum creatinine (78 vs 78 umol/l, p = 0.75). Further, there was no significant difference between the two groups in terms of LVEF, culprit vessel location, presence of multi-vessel CAD or post PCI TIMI flow. The presence of the key predictor, DM, was found to be associated with a significantly higher percentage of patients with RVD (45 vs 22 %, p = 0.03). Peak CK (1666.5 standard units/l, vs 2096 standard units/l, p = 0.25) and the presence of a previous MI (10 vs 1.7 %, p = 0.15) showed a trend towards a significant difference between the two groups. There was no significant difference in terms of infarct territory, presence of multi-vessel coronary artery disease and LVEF between the two groups.

### Logistic regression analyses

Table 5 displays the results of our logistic regression model. In this model, DM, presence of prior MI and Peak CK were included as predictors. DM remained a predictor for the presence of RVD after adjusting for other co-variates (p = 0.03). Further, the presence of DM was associated with an approximately threefold greater odds of RVD post STEMI (OR 2.78, 95 % CI 1.12, 6.87). No other predictor variables were independently associated with the presence of RVD in our cohort. The subgroup of diabetic patients with a HbAIC ≥7 % had an approximately fivefold greater odds of being associated with RVD (OR 5.58 (1.20, 25.78), p = 0.02). Our sensitivity analysis utilizing an RVEF cutoff of 47 % did not result in a significant change in our point estimate (OR 2.46 (95 % CI 1.02, 6.05)) (See Appendices 1, 2 for full results of the sensitivity analyses).

**Table 5:** Adjusted odds ratios of predictor variables for the outcome of presence or absence of RVD

Predictor	Odds ratio (95 % CI)	Test statistic	p value
Omnibus Likelihood Ratio [*x* ^2^ (df)]		9.46 (3)	0.02
Prior MI	4.67 (0.46, 47.03)	1.71	0.19
Peak CK in standard units/l	1.0 (0.99, 1.0)	1.39	0.24
Diabetes mellitus	2.78 (1.12, 6.87)	4.88	0.03

*df* degrees of freedom

## Discussion

32 (30.2 %) patients in our cohort had diabetes. With the exception of previous PCI and dyslipidemia, there were no significant differences between patients with and without diabetes in our cohort with regards to other cardiovascular risk factors, multi-vessel CAD or TIMI flow post PCI. Our results indicate that DM is an independent predictor for the presence of RVD post STEMI, conferring an approximate threefold greater odds of its presence. These results were robust across two definitions of RVD. Furthermore, higher levels of HbA1C were associated with a greater odds of the presence of RVD when compared to lower levels.

Emerging evidence suggests that the RV is an independent entity and its function is not necessarily dependent on left ventricular function. This has been highlighted by results of a number of recent studies that assessed ventricular function by CMR. Masci et al. performed CMR scanning on 242 consecutive patients post primary PCI for acute STEMI. They found that there was no significant difference in the mean LVEF between the group with (52 ± 9 %) and without (51 % ± 11) RV injury (p = 0.44) [24]. Similarly, Grothoff et al., studies 450 patients 1–4 days after primary PCI in STEMI. They also found that there was no significant difference in LVEF between the group with (48 ± 9 %) and without (50 ± 10 %) RV dysfunction [25]. These finding are consistent with our results showing no significant differences in LVEF between the RVD and the no RVD groups.

Right ventricular dysfunction has been shown to be an important predictor of cardiac events and mortality in multiple cardiac disease states. For example, a recent study reported that the presence of RVD, as measured by CMR derived RVEF, was an independent predictor of major adverse cardiac events in patients with heart failure with preserved ejection fraction [26]. Furthermore, RVD has been shown to be associated with increased morbidity and mortality post STEMI [8, 27–29]. Zehender et al. reported a higher incidence of major in-hospital complications in patients with right ventricular infarction vs. those without (64 vs 28 %, p < 0.001). Logistic-regression analysis showed that right ventricular infarction was an independent predictor of mortality (relative risk 7.7, 95 % CI (2.6–23)) [27]. Jacobs et al., in an analysis of patients in the SHOCK registry reported that in-hospital mortality was surprisingly not significantly different between those with predominantly right and left ventricular shock (53.1 vs 60.8 % respectively (p = 0.296)). Mehta et al. performed a meta-analysis of six studies (n = 1198) which demonstrated that RVD was associated with an increased risk of ventricular tachycardia or fibrillation (OR 2.7, 95 % CI 2.1–3.5), shock (OR 3.2, 95 % CI 2.4–3.5), and all-cause mortality (odds ratio [OR] 3.2, 95 % confidence interval [CI] 2.4–4.1) [28]. Cardiac magnetic resonance imaging is considered the reference standard for the assessment of right ventricular function [30–33]. RVD as measured by CMR has been shown to be an independent predictor of mortality. Larose et al., were the first group to show that RVD post STEMI as assessed by CMR was an independent predictor of mortality conferring an adjusted hazard ratio of 2.86 (p = 0.03) [31]. Recently, RVD as assessed *early* post STEMI was also reported to be an independent predictor of mortality [30]. Despite the clear prognostic importance of right ventricular infarction and dysfunction there have been no studies, to our knowledge, that have assessed the clinical predictors of right ventricular dysfunction post STEMI as determined by CMR. Anavekar et al. assessed RV function using echocardiographically determined fractional area of change (FAC). Their stated objective was to examine the relationship between RVD as measured by FAC and downstream cardiac outcomes. While they did not examine independent predictors of RVD, they did compare baseline characteristics of patients with and without RVD. They did not show a significant difference between the RVD and non RVD groups with regards to diabetes status [34]. Similarly, Zornoff et al. also aimed to assess the relationship between RVD as measured by echo derived FAC and downstream clinical outcomes. While the focus of the study was also not to determine independent predictors of RVD, they did compare baseline characteristics between those with and those without it. They also found that there was no significant difference between the RVD and the no RVD group with respect to DM status. Interestingly, there was a trend towards a significant difference as 24.1 % of patients with RVD had DM vs 17.5 % with no RVD (p = 0.18) [35]. A possible explanation for the discrepancy between our results and those of the two aforementioned papers lies in the method of assessing RV function. While some studies have shown good correlation between the RVEF and echo derived FAC [36], others have shown more moderate correlation. For example, one paper reported a correlation co-efficient of 0.34 for agreement between echo derived FAC and RVEF as calculated by CMR [37].

Multiple studies have shown that patients with diabetes have greater plaque burden and multivessel coronary artery disease [38, 39]. This observation is not new. In 1987 the thrombolysis and angioplasty in myocardial infarction trial (TAMI) was published. In that study, diabetics had a significantly higher incidence of multi-vessel CAD when compared to non-diabetics at the time of angiography [39]. More recently, Maffai et al. assessed 147 diabetic and 979 non-diabetic male patients who underwent coronary computed tomography angiography. They founds that patients with diabetes had a significantly higher plaque burden including a higher proportion of multi-vessel coronary artery disease [38]. However, the issue of plaque burden in patients with diabetes appears to be more complex then previously thought. A recent paper from Duce et al. looked at whole body magnetic resonance angiography and determined a standardized atheroma score (sas). Those with established cardiovascular disease without diabetes a higher whole body standardized atheroma score when compared to those with cardiovascular disease and diabetes. Further, only the former group was significantly different from the sas calculated from normal healthy volunteers. The authors were surprised by their results and opined that their results may indicate that cardiovascular events may occur at a lower atheroma burden in diabetes [40]. In our paper, there was no significant difference in terms of multi-vessel CAD between diabetics and non-diabetics. We feel that this may be due in part to the exclusion criteria for our study. We excluded patients who were referred for CABG after their angiogram. This was done in order to minimize potential complications during MRI scanning in those patients with potentially non-revascularized significant coronary lesions. We speculate that by doing so, we excluded many potential patients with diabetes who had multi-vessel CAD.

The issue of whether or not patients with diabetes have larger infarcts and subsequently lower LVEF is controversial. Alegria et al., reported on 1137 post STEMI patients from the CORE trial. They reported that patients with diabetes had a slightly lower median LVEF, as measured by gated equilibrium nuclear scanning, vs. those patients without diabetes (48 vs 51 %, p = 0.007) [41]. More recently and using MRI, Cassidy et al., reported that while some parameters of systolic and diastolic function were impaired in patients with diabetes, there was no significant difference in LVEF between diabetics and non-diabetics [42]. Further, Duce et al. reported no significant difference in MRI derived LVEF between those patients with manifest cardiovascular disease with and without diabetes [40]. In synthesizing this data, a reasonable conclusion is that there may be some difference between diabetics and non-diabetics in terms of LV function in those with coronary artery disease including post STEMI. However, this difference appears to be small, especially in contemporary cohorts receiving current standard of care treatment. This is consistent with the findings in our study.

Our results indicate that the presence of DM was an independent predictor for the presence of RVD post STEMI. Interestingly, no other major cardiovascular risk factors were independently associated with the presence of RVD. In the absence of a significant difference in multi-vessel macrovascular disease or procedural success between the two groups, a possible explanation for this may lie in the diabetic microvasculature as well as the blood supply to the RV. The physiology of the RV confers relative protection against ischemia when compared to the left ventricle. The RV has a dual blood supply, is perfused during both systole and diastole, and is heavily perfused by microvascular collateral vessels [8, 43, 44]. Patients with DM have extensive coronary microvascular dysfunction and disease [12, 13, 45]. It is possible that this predilection for microvascular disease attenuates some of the natural protective effects of the RV and predisposes to the presence of RVD post STEMI.

Further, our results indicate that patients with HbA1C ≥7 % were more likely to develop RVD vs. those with HbA1C <7 %. This supports previous work that has shown that chronic hyperglycemia is associated with more microvascular dysfunction [11, 12]. It also supports the idea that the underlying reason for the increase in frequency of RVD in our cohort may involve diabetic microvascular disease.

These results are important because, if replicated in future larger studies, they can help risk stratify patients post STEMI with respect to their probability of developing RVD. Since multiple studies have shown that RVD is an important prognostic marker of mortality post MI and since patient management is different in those with and without right ventricular dysfunction, having an ability to stratify post-MI patients according to their risk for developing RVD may influence patient management.

### Future directions

In order to test our hypothesis that microvascular dysfunction is the mechanism underlying the increased likelihood of diabetic patients being associated with RVD, future studies should directly measure microvascular function (for example by BOLD myocardial imaging) and assess its relationship with downstream development of RVD.

### Limitations

This study needs to be interpreted in the context of its limitations. First, this is an observational study and while we tried to account for all clinically relevant confounders in our logistic regression model, there is the possibility of potentially clinically meaningful unmeasured confounders that were not incorporated into our model. Second, there are different definitions in the literature for what constitutes RVD. In order to try and overcome this, we performed a sensitivity analysis using another definition of RVD and found that this did not significantly affect our results. Third, the number of patients in our study was relatively small. Thus, significant differences between the RVD and no RVD groups that we may not have detected cannot be fully excluded. For example, considering the low number of females, a potential effect of gender on the presence of RVD cannot be ruled out. Fourth, there was no pre-imaging of patients and thus we could not comment on diabetes being a risk factor in the development of RVD but rather only in the presence of it. Finally, a normal HbA1C does not preclude diabetes thus potentially confounding our results.

## Conclusions

Our results indicate that the presence of DM is an independent predictor for the presence of right ventricular dysfunction post STEMI, conferring an approximately 3x-fold greater odds of its presence. No other major cardiovascular risk factor was associated with the presence of RVD post STEMI. These results may help to identify patients post STEMI who are at risk for the presence of RVD. Further research is required to understand the pathophysiology behind the presence of RVD so that potential therapeutic strategies can be identified.

## Appendix 1

See Table 6.

**Table 6:** Adjusted odds ratios of predictor variables for the outcome of presence or absence of RVD (with RVEF cut-off of 47 %)

Predictor	Odds ratio (95 % CI)	p value
Prior MI	1.10 (0.16, 7.53)	0.92
Peak CK in standard units/l	1.00 (0.99, 1.00)	0.85
Diabetes mellitus	2.46 (1.02, 6.05)	0.04

## Appendix 2

See Table 7.

**Table 7:** Adjusted odds ratios of predictor variables for the outcome of presence or absence of RVD in those patients with an HbA1C ≥7

Predictor	Odds ratio (95 % CI)	p value
Prior MI	2.48 (0.10, 64.48)	0.58
Peak CK in standard units/l	1.00 (0.99, 1.00)	0.32
Diabetes mellitus	5.58 (1.20, 25.78)	0.02

## Abbreviations

CAD: coronary artery disease
CMR: cardiac magnetic resonance imaging
eGFR: estimated glomerular filtration rate
MI: myocardial infarction
MRI: magnetic resonance imaging
RVD: right ventricular dysfunction
RVMI: right ventricular myocardial infarction
SCMR: society for cardiovascular magnetic resonance
TIMI: thrombolysis in myocardial infarction

## References

[1] Organization WH. World Health Organization Diabetes Programme. 2015. http://www.who.int/diabetes/en/.

[2] Malmberg K, Yusuf S, Gerstein HC, Brown J, Zhao F, Hunt D (2000). Impact of diabetes on long-term prognosis in patients with unstable angina and non-Q-wave myocardial infarction: results of the OASIS (Organization to assess strategies for ischemic syndromes) Registry. Circulation.

[3] Murcia AM, Hennekens CH, Lamas GA, Jimenez-Navarro M, Rouleau JL, Flaker GC (2004). Impact of diabetes on mortality in patients with myocardial infarction and left ventricular dysfunction. Arch Intern Med.

[4] Tocci G, Ferrucci A, Guida P, Avogaro A, Comaschi M, Corsini A (2011). Impact of diabetes mellitus on the clinical management of global cardiovascular risk: analysis of the results of the evaluation of final feasible effect of control training and ultra sensitization (EFFECTUS) educational program. Clin Cardiol.

[5] Tardif JC, L’Allier PL, Fitchett DH (2013). Management of acute coronary syndromes. Can J Diabetes..

[6] Simon MA (2013). Assessment and treatment of right ventricular failure. Nat Rev Cardiol..

[7] Fang ZY, Prins JB, Marwick TH (2004). Diabetic cardiomyopathy: evidence, mechanisms, and therapeutic implications. Endocr Rev.

[8] Ondrus T, Kanovsky J, Novotny T, Andrsova I, Spinar J, Kala P (2013). Right ventricular myocardial infarction: from pathophysiology to prognosis. Exp Clinical Cardiol..

[9] Haddad F, Hunt SA, Rosenthal DN, Murphy DJ (2008). Right ventricular function in cardiovascular disease, part I: anatomy, physiology, aging, and functional assessment of the right ventricle. Circulation.

[10] Klein R, Klein BE, Moss SE, Cruickshanks KJ (1994). Relationship of hyperglycemia to the long-term incidence and progression of diabetic retinopathy. Arch Intern Med.

[11] Bash LD, Selvin E, Steffes M, Coresh J, Astor BC (2008). Poor glycemic control in diabetes and the risk of incident chronic kidney disease even in the absence of albuminuria and retinopathy: atherosclerosis risk in communities (ARIC) study. Arch Intern Med.

[12] Di Carli MF, Janisse J, Grunberger G, Ager J (2003). Role of chronic hyperglycemia in the pathogenesis of coronary microvascular dysfunction in diabetes. J Am Col Cardiol..

[13] Camici PG, Crea F (2007). Coronary microvascular dysfunction. N Engl J Med.

[14] O’Gara PT, Kushner FG, Ascheim DD, Casey DE, Chung MK, de Lemos JA (2013). 2013 ACCF/AHA guideline for the management of ST-elevation myocardial infarction: a report of the American College of Cardiology Foundation/American Heart Association Task Force on Practice Guidelines. J Am Col Cardiol..

[15] O’Gara PT, Kushner FG, Ascheim DD, Casey DE, Chung MK, de Lemos JA (2013). 2013 ACCF/AHA guideline for the management of ST-elevation myocardial infarction: a report of the American College of Cardiology Foundation/American Heart Association Task Force on Practice Guidelines. Circulation.

[16] Schulz-Menger J, Bluemke DA, Bremerich J, Flamm SD, Fogel MA, Friedrich MG (2013). Standardized image interpretation and post processing in cardiovascular magnetic resonance: Society for Cardiovascular Magnetic Resonance (SCMR) board of trustees task force on standardized post processing. J Cardiovasc Magn Reson.

[17] Roifman I, Zia MI, Zavodni A, Wolff R, Ghugre NR, Leber AW (2014). Evolution of right ventricular function post-acute ST elevation myocardial infarction. J Magn Reson Imaging.

[18] Pavlicek M, Wahl A, Rutz T, de Marchi SF, Hille R, Wustmann K (2011). Right ventricular systolic function assessment: rank of echocardiographic methods vs cardiac magnetic resonance imaging. Eur J..

[19] Wahl A, Praz F, Schwerzmann M, Bonel H, Koestner SC, Hullin R (2011). Assessment of right ventricular systolic function: comparison between cardiac magnetic resonance derived ejection fraction and pulsed-wave tissue Doppler imaging of the tricuspid annulus. Int J Cardiol.

[20] Movahed MR, Milne N (2008). Poor correlation between left and right ventricular ejection fractions in patients with normal ventricular function. Exp Clin Cardiol..

[21] Yusuf S, Hawken S, Ounpuu S, Dans T, Avezum A, Lanas F (2004). Effect of potentially modifiable risk factors associated with myocardial infarction in 52 countries (the INTERHEART study): case-control study. Lancet.

[22] Yoshida M, Mita T, Yamamoto R, Shimizu T, Ikeda F, Ohmura C (2012). Combination of the Framingham risk score and carotid intima-media thickness improves the prediction of cardiovascular events in patients with type 2 diabetes. Diabetes Care.

[23] Treeprasertsuk S, Leverage S, Adams LA, Lindor KD (2012). St Sauver J, Angulo P. The Framingham risk score and heart disease in nonalcoholic fatty liver disease. Liver Int..

[24] Masci PG, Francone M, Desmet W, Ganame J, Todiere G, Donato R (2010). Right ventricular ischemic injury in patients with acute ST-segment elevation myocardial infarction: characterization with cardiovascular magnetic resonance. Circulation.

[25] Grothoff M, Elpert C, Hoffmann J, Zachrau J, Lehmkuhl L, de Waha S (2012). Right ventricular injury in ST-elevation myocardial infarction: risk stratification by visualization of wall motion, edema, and delayed-enhancement cardiac magnetic resonance. Circ Cardiovasc Imaging..

[26] Aschauer S, Kammerlander AA, Zotter-Tufaro C, Ristl R, Pfaffenberger S, Bachmann A (2016). The right heart in heart failure with preserved ejection fraction: insights from cardiac magnetic resonance imaging and invasive haemodynamics. Eur J Heart Fail.

[27] Zehender M, Kasper W, Kauder E, Schonthaler M, Geibel A, Olschewski M (1993). Right ventricular infarction as an independent predictor of prognosis after acute inferior myocardial infarction. N Engl J Med.

[28] Mehta SR, Eikelboom JW, Natarajan MK, Diaz R, Yi C, Gibbons RJ (2001). Impact of right ventricular involvement on mortality and morbidity in patients with inferior myocardial infarction. J Am Col Cardiol..

[29] Jacobs AK, Leopold JA, Bates E, Mendes LA, Sleeper LA, White H (2003). Cardiogenic shock caused by right ventricular infarction: a report from the shock registry. J Am Col Cardiol..

[30] Miszalski-Jamka T, Klimeczek P, Tomala M, Krupinski M, Zawadowski G, Noelting J (2010). Extent of RV dysfunction and myocardial infarction assessed by CMR are independent outcome predictors early after STEMI treated with primary angioplasty. JACC Cardiovasc Imaging..

[31] Larose E, Ganz P, Reynolds HG, Dorbala S, Di Carli MF, Brown KA (2007). Right ventricular dysfunction assessed by cardiovascular magnetic resonance imaging predicts poor prognosis late after myocardial infarction. J Am Col Cardiol..

[32] Markiewicz W, Sechtem U, Higgins CB (1987). Evaluation of the right ventricle by magnetic resonance imaging. Am Heart J.

[33] Baur LH (2008). Magnetic resonance imaging: the preferred imaging method for evaluation of the right ventricle. Int J Cardiovasc Imaging.

[34] Anavekar NS, Skali H, Bourgoun M, Ghali JK, Kober L, Maggioni AP (2008). Usefulness of right ventricular fractional area change to predict death, heart failure, and stroke following myocardial infarction (from the VALIANT ECHO study). Am J Cardiol.

[35] Zornoff LA, Skali H, Pfeffer MA, St John Sutton M, Rouleau JL, Lamas GA (2002). Right ventricular dysfunction and risk of heart failure and mortality after myocardial infarction. J Am Col Cardiol..

[36] Anavekar NS, Gerson D, Skali H, Kwong RY, Yucel EK, Solomon SD (2007). Two-dimensional assessment of right ventricular function: an echocardiographic-MRI correlative study. Echocardiography..

[37] Kjaergaard J, Petersen CL, Kjaer A, Schaadt BK, Oh JK, Hassager C (2006). Evaluation of right ventricular volume and function by 2D and 3D echocardiography compared to MRI. Eur J Echocardiogr..

[38] Maffei E, Seitun S, Nieman K, Martini C, Guaricci AI, Tedeschi C (2011). Assessment of coronary artery disease and calcified coronary plaque burden by computed tomography in patients with and without diabetes mellitus. Eur Radiol.

[39] Topol EJ, Califf RM, Kereiakes DJ, George BS (1987). Thrombolysis and Angioplasty in Myocardial Infarction (TAMI) trial. J Am Col Cardiol..

[40] Duce SL, Weir-McCall JR, Gandy SJ, Matthew SZ, Cassidy DB, McCormick L (2015). Cohort comparison study of cardiac disease and atherosclerotic burden in type 2 diabetic adults using whole body cardiovascular magnetic resonance imaging. Cardiovasc Diabetol..

[41] Alegria JR, Miller TD, Gibbons RJ, Yi QL, Yusuf S (2007). Infarct size, ejection fraction, and mortality in diabetic patients with acute myocardial infarction treated with thrombolytic therapy. Am Heart J.

[42] Cassidy S, Hallsworth K, Thoma C, MacGowan GA, Hollingsworth KG, Day CP (2015). Cardiac structure and function are altered in type 2 diabetes and non-alcoholic fatty liver disease and associate with glycemic control. Cardiovasc Diabetol..

[43] Bonanad C, Ruiz-Sauri A, Forteza MJ, Chaustre F, Minana G, Gomez C (2013). Microvascular obstruction in the right ventricle in reperfused anterior myocardial infarction. Macroscopic and pathologic evidence in a swine model. Thromb Res.

[44] Voelkel NF, Quaife RA, Leinwand LA, Barst RJ, McGoon MD, Meldrum DR (2006). Right ventricular function and failure: report of a National Heart, Lung, and Blood Institute working group on cellular and molecular mechanisms of right heart failure. Circulation.

[45] Tomai F, Ribichini F, Ghini AS, Ferrero V, Ando G, Vassanelli C (2005). Elevated C-reactive protein levels and coronary microvascular dysfunction in patients with coronary artery disease. Eur Heart J.

